# Vulnerability Imposed by Diet and Brain Trauma for Anxiety-Like Phenotype: Implications for Post-Traumatic Stress Disorders

**DOI:** 10.1371/journal.pone.0057945

**Published:** 2013-03-06

**Authors:** Ethika Tyagi, Rahul Agrawal, Yumei Zhuang, Catalina Abad, James A. Waschek, Fernando Gomez-Pinilla

**Affiliations:** 1 Department of Integrative Biology and Physiology, University of California Los Angeles, Los Angeles, California, United States of America; 2 Department of Neurosurgery, University California Los Angeles Brain Injury Research Center, Los Angeles, California, United States of America; 3 Semel Institute for Neuroscience and Human Behavior, Department of Psychiatry, David Geffen School of Medicine at University of California Los Angeles, Los Angeles, California, United States of America; University of Pittsburgh, United States of America

## Abstract

Mild traumatic brain injury (mTBI, cerebral concussion) is a risk factor for the development of psychiatric illness such as posttraumatic stress disorder (PTSD). We sought to evaluate how omega-3 fatty acids during brain maturation can influence challenges incurred during adulthood (transitioning to unhealthy diet and mTBI) and predispose the brain to a PTSD-like pathobiology. Rats exposed to diets enriched or deficient in omega-3 fatty acids (n-3) during their brain maturation period, were transitioned to a western diet (WD) when becoming adult and then subjected to mTBI. TBI resulted in an increase in anxiety-like behavior and its molecular counterpart NPY1R, a hallmark of PTSD, but these effects were more pronounced in the animals exposed to n-3 deficient diet and switched to WD. The n-3 deficiency followed by WD disrupted BDNF signaling and the activation of elements of BDNF signaling pathway (TrkB, CaMKII, Akt and CREB) in frontal cortex. TBI worsened these effects and more prominently in combination with the n-3 deficiency condition. Moreover, the n-3 deficiency primed the immune system to the challenges imposed by the WD and brain trauma as evidenced by results showing that the WD or mTBI affected brain IL1β levels and peripheral Th17 and Treg subsets only in animals previously conditioned to the n-3 deficient diet. These results provide novel evidence for the capacity of maladaptive dietary habits to lower the threshold for neurological disorders in response to challenges.

## Introduction

The balance between brain health and disease is often unpredictable and likely dependent on poorly understood vulnerability factors acquired particularly during early life [1], [2]. Changes in lifestyles and living conditions associated with globalization impose unprecedented new challenges for the etiology of neurological disorders. Dietary factors are surfacing as strong modulators of brain plasticity [3], [4] with the capacity to alter the course of brain disorders. We have embarked in studies to understand how dietary omega-3 fatty acid during brain maturation influences the capacity of the brain to cope with later challenges. Among the challenges, adoption of unhealthy dietary habits is becoming increasingly common in the modern society, and this transition may act as a vulnerability factor for neurological disorders.

In particular, post-traumatic stress disorder (PTSD) has emerged as a mysterious condition in which individuals exposed to trauma develop high levels of anxiety and inability to cope with routine living conditions [5]. Among of the few clues, now we know that a large number of victims exposed to episodes of brain trauma develop PTSD, likely influenced by some pre-existing conditions. In the current study, we present evidence that switching from omega-3 fatty acids deficiency during brain formation to a diet high in calories in adult life weaken the substrates for brain plasticity and predispose the brain to elevated anxiety-like behavior.

The omega-3 fatty acid docosohexaenoic acid (DHA) is structured in neuronal plasma membranes and crucial for neuronal signaling. Diet is the only source of DHA for the brain and subnormal content of DHA has been associated with mood disorders in humans [6], i.e., increased risk of suicide in a population exposed to trauma [7]. Dietary DHA has been shown to protect against cognitive impairment following brain trauma in rodents [8], [9]. Consumption of DHA is below recommended levels while the consumption of high fat and high sugar is on the raise in the western society [10], and this hardship has been attributed to increased incidence of psychiatric disorders [11], [12]. Therefore we embarked in studies to investigate how dietary DHA during brain formation could help resist the challenge imposed by transitioning to a western diet (WD) during adulthood and the outcome of brain trauma.

Given that TBI is a risk factor for PTSD, we sought to explore the effects of dietary switching on crucial mechanisms underlying the development of anxiety-like disorders in animals exposed to concussive injury. It has been previously shown that this type of concussive injury predispose to anxiety and fear with some similarities to a PTSD-like condition [13]. Individuals affected with PTSD exhibit altered expression of molecules involved in immune activation [14] while brain trauma alters release of cytokines such as interleukin-1β (IL-1β) and IL-10 [15]. Given that n-3 fatty acids have immunomodulatory capacity [16], we studied their effects on crucial parameters of peripheral and central immune function in our paradigm. We also assessed brain-derived neurotrophic factor (BDNF) based on its recognized role on anxiety and depression [17] and the fact that it is reduced in the plasma of PTSD victims [18].

## Materials and Methods

### Animals and Diets

Female Sprague–Dawley rats (250–280 gm) were obtained from Charles River Laboratories (Wilmington, MA) on 2nd day of pregnancy and were kept under standard housing condition (22–24°C) with 12-h light/dark cycle in two dietary groups. One group of pregnant females was fed an omega-3 fatty acid adequate diet (n-3 diet) and the other group was fed an omega-3 fatty acid deficient diet (n-3 def) through gestation and lactation. The diets were provided in powder in a bowl and animals had free access to food and water. The custom diets used were based on the composition of American Institute of Nutrition Diet and prepared commercially (Dyets Inc. Bethlehem, PA) as previously described [19]. Both diets had the similar basal macronutrients, vitamins, minerals and basal fats (hydrogenated coconut and safflower oils) (Table 1). The total fat content in both diets was ∼10% (w/w) but the only difference between two diets was the amount of n-3 fatty acids, which was achieved by adding 0.5% of flaxseed (source of 18:3*n*-3; ALA) and 1.2% of docosahexaenoic acid (Procured from Nordic Naturals, Inc., Watsonville, CA, USA as ProDHA capsule) to the n-3 diet.

**Table 1:** Composition of experimental diets.

Ingredient	Amount (g/100 g diet)	
	n-3 adq	n-3 def
Alacid 710, acid casein	20	20
Cornstarch	15	15
Sucrose	10	10
Dextrose	19	19.9
Maltose-dextrin	15	15
Cellulose	5	5
Salt-mineral mix	3.5	3.5
Vitamin mix	1	1
L-cystine	0.3	0.3
Choline bitartrate	0.25	0.25
TBHQ	0.002	0.002
**Fat sources:**		
Hydrogenated coconut oil	7.45	8.1
Safflower oil	1.77	1.9
Flaxseed oil	0.48	–
DHA^a^	1.2	–

Note: Dashes indicate that component was not added.

a Procured from Nordic Naturals, Inc., Watsonville, CA, USA as ProDHA capsule that contains 45% (w/w) DHA and 9% (w/w) EPA.

### Experimental Design

**Figure 1:**
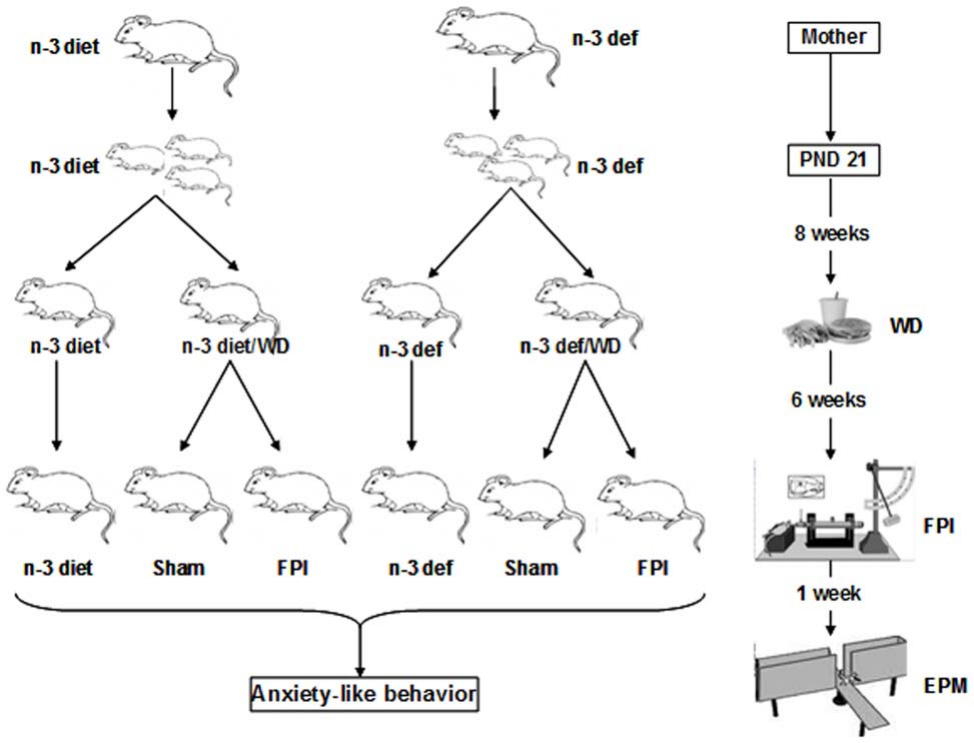
Schematic timeline representing experimental design to study the impact of dietary transition on anxiety-like behavior in response to traumatic brain injury. Sprague Dawley female rats were fed on omega-3 adequate diet (n-3 diet; left) or omega-3 deficient diet (n-3 def; right) from 2nd day of pregnancy. On postnatal day (PND) 21, pups from each group were weaned to the same diet as their dams for 8 weeks and only male pups from each dietary group was used in the study. Further, offspring from each dietary group (n-3 diet, n = 10; n-3 def, n = 10) were switched to western diet (WD) up to 15 weeks and after 6 weeks of WD, half of these animals from each group were subjected to the fluid percussion injury (FPI). Remaining pups (n-3 diet, n = 5; n-3 def, n = 5) were maintained on the same diet for the entire 15 weeks duration. After 1 week of FPI, all animals were subjected to the elevated plus maze (EPM) test for anxiety-like behavior and then sacrificed immediately for tissue collection.

A total of 30 male pups were randomly selected for behavioral, molecular and fatty acid studies, and each pup within a dietary group was selected from a different litter; therefore, one animal from each litter was tested in each dietary group. From postnatal day (PND) 21, the pups from each group (n-3 diet, n = 15; n-3 def, n = 15) were weaned to the same diet as their dams for 8 weeks and only male pups from each dietary group were used in the study. Further, offspring from each dietary group (n-3 diet, n = 10; n-3 def, n = 10) were transitioned to western diet (WD; D12079B, Research Diets Inc. NJ, USA) upto 15 weeks. Both n-3 diets have 10% (w/w) sugar and 10% (w/w) total fat, however, WD contains 34% (w/w) sugar and 21% (w/w) total fat. After 6 weeks of dietary transition, half of these animals from each group were subjected to the fluid percussion injury (FPI). Remaining pups (n-3 diet, n = 5; n-3 def, n = 5) were maintained on the same diet for the entire 15 weeks duration (Figure 1). Additionally, a separate cohort of pups (n = 3 per group, n-3 diet or n-3 def) was also selected from the same litters (that were used for molecular studies) in each dietary group for immunofluoresence studies. Therefore, 36 animals were used in total for whole study. All experiments were performed in accordance with the United States National Institutes of Health Guide for the Care and Use of Laboratory Animals and were approved by the University of California at Los Angeles Chancellor’s Animal Research Committee. All efforts were made to minimize suffering and number of animals used.

### Fluid Percussion Injury (FPI)

After 6 weeks of dietary transition, FPI was performed as previously described [8]. In brief, with the aid of a microscope (Wild, Heerbrugg, Switzerland) a 3.0-mm-diameter craniotomy was made over the left parietal cortex, 3.0 mm posterior to bregma and 6.0 mm lateral (left) to the midline with a high-speed drill (Dremel, Racine, WI). A plastic injury cap was placed over the craniotomy with silicone adhesive and dental cement. When the dental cement hardened, the cap was filled with 0.9% saline solution. Anesthesia was discontinued and the injury cap was attached to the fluid percussion device. At the first sign of hind-limb withdrawal to a paw pinch, a mild fluid percussion pulse (1.5 atm) was administered to the epidural space. Immediately upon responding to a paw pinch, anesthesia was restored and the skull was sutured. Neomycin was applied on the suture and the rats were placed in a heated recovery chamber before being returned to their cages. Sham animals underwent an identical procedure with the exception of the lesion.

### Elevated Plus Maze (EPM)

After 1 week of FPI, EPM test was performed according to the [20]. Briefly the EPM apparatus made of laminated wood consisted of two opposing open arms (10×50 cm) and two opposing close arms (10×50 cm with 30 cm high walls). The maze was placed 60 cm above the floor. Whit curtains surrounded the maze and behavior was recorded by an overhead video camera over a period of 5 min. Each rat was placed in the middle of the maze facing the open arm away from the experimenter. The time spent and the number of entries in each arm was measured using AnyMaze video tracking software (San Diego Instruments, San Diego, CA). The total number of times rats entered in arms during the EPM test was calculated to account for differences in general motor activity in the maze.

### Tissue Collection

After finishing the EPM test (i.e, on day 7 after injury), the animals were killed by decapitation and the fresh brains were collected, frozen in dry ice and stored at −70°C until use.

### Preparation of Cell Suspensions and Flow Cytometry Staining

At the time of decapitation the spleen was removed and suspensions were prepared as previously described [21]. The tissue was first teased in ice cold phosphate-buffered saline (PBS) with forceps on 0.45μ size mesh and centrifuged at 1000×g for 10 min at 4°C. Red blood cell lysis buffer (150 mM NH_4_Cl, 9 mM KHCO_3_, 0.12 mM EDTA) was added to the pellet, mixed gently and incubated for 5 min on ice. After adding ice cold PBS, centrifuged at 1000×g at 4°C for 10 min. The cell suspension was then washed and resuspended in PBS supplemented with 1% BSA. For stimulation, 5×10^5^ cells were incubated with complete RPMI medium 1640 (with 2% FBS; HyClone) containing PMA (50 ng/mL; Sigma), ionomycin (1 µg/mL; Sigma), and brefeldin (3 µg/mL; eBioscience) for 4 hrs at 37°C with 5% CO_2_. After incubation for flow cytometric analysis, aliquots of 1×10^5^ cells/tube were stained with saturating amounts of CD4/fluorescein isoththiocyanate (FITC) and/or IL-17/peridinin chlorophyll protein (PerCP) conjugated antibodies (eBiosciences, USA), according to the manufacturer's instructions. For nuclear staining, cells were fixed with 2% PFA for 15 min at 4°C and permeabilized by incubating with 0.2% Tween-20 for 15 min at 37°C, and then incubated with CD25/phycoerythrin (PE) and FoxP3/allophycocyanin (APC) specific antibodies (eBiosciences, USA). After incubation the cell suspension was washed and resuspended in PBS supplemented with 1% BSA and was analyzed with a FACSCalibur flow cytometer.

### Cytokines Estimation

The frontal cortical tissues were homogenized in ice-cold Tris buffer (pH 7.2, 4°C, 10% w/v) containing 50 mM Tris, 1 mM EDTA, 6 mM MgCl_2,_ 1 mM phenylmethylsulfonylfluoride (PMSF), 10 µg/ml aprotinin, 1 µg/ml leupeptin, 0.1 mM benzethonium chloride and 0.5 mM sodium vanadate. After homogenization, samples were sonicated for 10 s using an ultrasonic processor (Cole Parmer, USA) at a setting of 5, and then centrifuged at 20,800 g for 20 min at 4°C. Supernatants were collected for cytokines estimation using ELISA kits (R&D Systems) as per manufacturer instructions.

### Immunobloting

The frontal cortical tissues were homogenized in a lysis buffer containing 137 mM NaCl, 20 mM Tris–HCl pH 8.0, 1% NP40, 10% glycerol, 1 mM phenylmethylsulfonylfluoride (PMSF), 10 µg/ml aprotinin, 1 µg/ml leupeptin, 0.1 mM benzethonium chloride, 0.5 mM sodium vanadate. The homogenates were then centrifuged, the supernatants were collected and total protein concentration was determined according to MicroBCA procedure (Pierce, IL, USA), using bovine serum albumin (BSA) as standard. Briefly, protein samples were separated by electrophoresis on 10% polyacrylamide gel and electrotransferred to a PVDF membrane (Millipore, Bedford, MA). Non-specific binding sites were blocked in Tris-buffered saline (TBS), pH 7.6, containing 5% non-fat dry milk. Membranes were rinsed in buffer (0.05% Tween-20 in TBS) and then incubated with anti-NPY1R (1∶1000; Alpha Diagnostic Intl. Inc., TX, USA) anti-BDNF, anti-pTrk, anti-Trk, anti-pCaMKII, anti-CaMKII and anti-actin, (1∶500; Santa Cruz Biotechnology, Santa Cruz, CA, USA), anti-pCREB, anti-CREB, (1∶1000, Millipore, MA, USA), anti-pAkt, Anti-Akt, (1∶1000; Cell signaling technology, MA, USA) followed by anti-rabbit or anti goat IgG horseradish peroxidase-conjugate (1∶10,000; Santa Cruz Biotechnology). After rinsing with buffer, the immunocomplexes were visualized by chemiluminescence using the ECL kit (Amersham Pharmacia Biotech Inc., Piscataway, NJ, USA) according to the manufacturer's instructions. The signals were digitally scanned and then quantified using ImageJ software. Data were standardized according to actin values.

### Immunofluorescence

Additional rats from n-3 and n-3 deficient diet were anesthetized with isoflurane, then intracardially perfused with 0.1 M PBS (pH 7.4) followed by 4% paraformaldehyde (PFA) in 0.1 M phosphate buffer (pH 7.4) and 30% sucrose in 4% PFA. Serial coronal brain sections (10 µm) were cut on a cryostat, and were mounted on Superfrost slides and non-specific binding was blocked by incubating the sections with PBS solution containing 3% BSA and 0.3% Triton X-100 for 2 h at room temperature. Sections were then incubated with appropriate dilutions of the primary antibody (CaMKII, Santa Cruz Biotechnology and NPY1R, Alpha Diagnostic Intl. Inc.; 1∶250) in PBS solution containing 1% BSA and 0.3% Triton X-100 at 4°C overnight in a humidified chamber. After thorough washing with PBS, the sections were incubated with appropriate dilutions of fluorescent mouse or rabbit secondary antibodies (FITC; 1∶1000, Cy3; 1∶4000) for 1 h at room temperature. Slides were washed in PBS and mounted using aquamount. Negative controls were performed by omission of the primary antibody. The results of immunohistochemistry controls were negative as no staining was observed in cell structures. The staining was visualized under Zeiss microscope (Zeiss Imager.Z1) using the Axiovision 4.6 software.

### Fatty Acid Analysis

Fatty acids were measured by gas chromatography in cerebral cortical tissue-extracted lipids. For extraction of lipids, frozen tissues were homogenized in chloroform/methanol (2∶1 vol/vol), containing 50 µg/ml of butylated hydroxytoluene to prevent lipid oxidation during the procedure. Tricosanoic acid methylester (C23:0) was used as an internal standard. Tissues were grounded to powder under liquid nitrogen and subjected to extraction of total lipids. Fatty acid methylation was done by heating at 90°C for 1 hr under 14% (w/v) boron trifluoride–methanol reagent.

Extracted lipids were analyzed on Clarus 500 gas chromatograph (GC; PerkinElmer) with a built-in Autosampler. An Elite-WAX column (60 m, 0.32-mm internal diameter, PerkinElmer) was used, with hydrogen as the carrier gas. GC oven temperature was initially held at 140 °C for 2 min and raised with a gradient of 5 °C min-1 until 250 °C and held for 10 min. The total run time is 34 min. The injector and detector were maintained at 250 and 300 °C, respectively. A 1 µl sample of Fatty acid methyl esters (FAME) was injected in split injection mode with a 100∶1 split ratio. Peaks of fatty acids were identified and quantified by comparison with standards Supelco 37-component FAME Mix (Sigma-Aldrich, MO, USA) and GLC reference standard 682 (Nu-Chek Prep, Inc. MN, USA).

### Statistical Analysis

The results are expressed as mean ± standard error of the mean (SEM). EPM, protein and fatty acid data were analyzed by two-way ANOVA [(diet: n-3 diet vs. n-3 def) and (treatment: None vs. WD vs. WD/FPI)] and post-hoc analyses were conducted using Newman-Keuls comparisons. Student’s (unpaired) t test was used to analyze the data of immune markers. The p-value <0.05 was considered as statistically significant. Analysis of correlation (linear regression) was performed on individual samples to evaluate the association between variables.

## Results

### Anxiety-like behavior after Adult Brain Trauma

**Figure 2:**
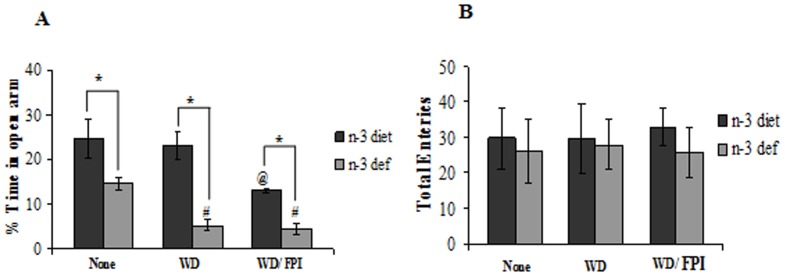
Effect of dietary alteration on anxiety-like behavior after TBI. Percentage of (**A**) time spent in open arm and (**B**) total entries in elevated plus maze (EPM) test in groups fed with omega-3 (n-3 diet) or omega-3 deficient (n-3 def) diet, transitioned to western diet (n-3 diet/WD and n-3 def/WD) and subjected to fluid percussion injury after diet transition (n-3 diet/WD/FPI and n-3 def/WD/FPI). Values are expressed as mean ± SEM. ^#^p<0.05 Vs n-3 def, ^@^p<0.05 Vs n-3 diet, ^*^p<0.05 Vs their respective n-3 diet group by ANOVA (two-way) and Newman–Keuls post-hoc test.

We assessed the impact of dietary transition as determinant factor for the outcome of mild traumatic brain injury (mTBI) on anxiety-like behavior. A two-way ANOVA analysis (diet vs. treatment) showed a significant effect of diet (F_1,24_ = 41.46, p<0.01) and treatment (F_2,24_ = 10.89, p<0.01) on the time spent in the open arm of the elevated plus maze (EPM). Dietary n-3 deficiency resulted in anxiety-like behavior as reflected by a significant reduction in time spent in open arms of EPM, which was further aggravated after transition to the WD (Figure 2A). Transitioning to WD did not affect the time spent in open arm in the n-3 adequate group, but this was reduced by subsequent TBI. TBI was not effective to further reduce the already lowered time spent in open arms in the WD group switched from n-3 deficient diet. There was no difference in total number of entries among all the groups (Figure 2B) ruling out a potential interference of motor activity on anxiety like behavior.

### Molecules Associated with Anxiety-like behavior: Role of NPY-1R

**Figure 3:**
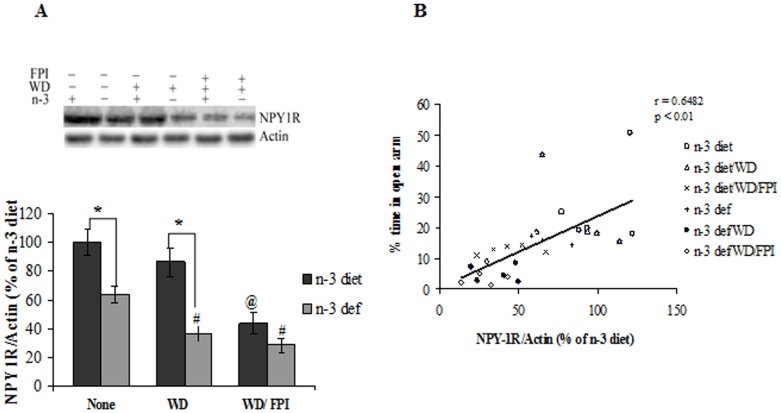
Influence of dietary alteration and TBI on molecules associated with anxiety-like behavior. Protein levels of (**A**) NPY1R and (**B**) correlation analysis of NPY1R with anxiety-like behavior in groups fed with omega-3 (n-3 diet) or omega-3 deficient (n-3 def) diet, transitioned to western diet (n-3 diet/WD and n-3 def/WD) and subjected to fluid percussion injury after diet transition (n-3 diet/WD/FPI and n-3 def/WD/FPI). Values are expressed as mean ± SEM. ^#^p<0.05 Vs n-3 def, ^@^p<0.05 Vs n-3 diet, ^*^p<0.05 Vs their respective n-3 diet group by ANOVA (two-way) and Newman–Keuls post-hoc test.

We assessed levels of the Neuropeptide-Y1 Receptor (NPY1R) in the frontal cortex based on the demonstrated role of NPY on anxiety-like behavior [22]. There was a significant effect of diet (F_1,24_ = 30.62, p<0.01) and treatment (F_2,24_ = 18.82, p<0.01) on the levels of NPY1Rs. We found that the dietary n-3 deficiency reduced NPY1R levels and switching to the WD reduced levels even further. However, prior exposure to dietary n-3 supplementation preserved the levels of NPY1R in animals transitioned to WD. Notably, TBI reduced the levels of NPY1R beyond the reduction elicited by the switching to WD in the animals belonging to n-3 groups, however, the effects were more pronounced in WD rats previously exposed to the n-3 deficient diet (Figure 3A). There was a positive correlation between NPY1R levels and percentage time spent in open arm (r = 0.6482, p<0.01), which is consistent with an involvement of NPY1R in anxiety-like behavior (Figure 3B).

### Molecules Associated with Synaptic Plasticity and Brain Trauma

**Figure 4:**
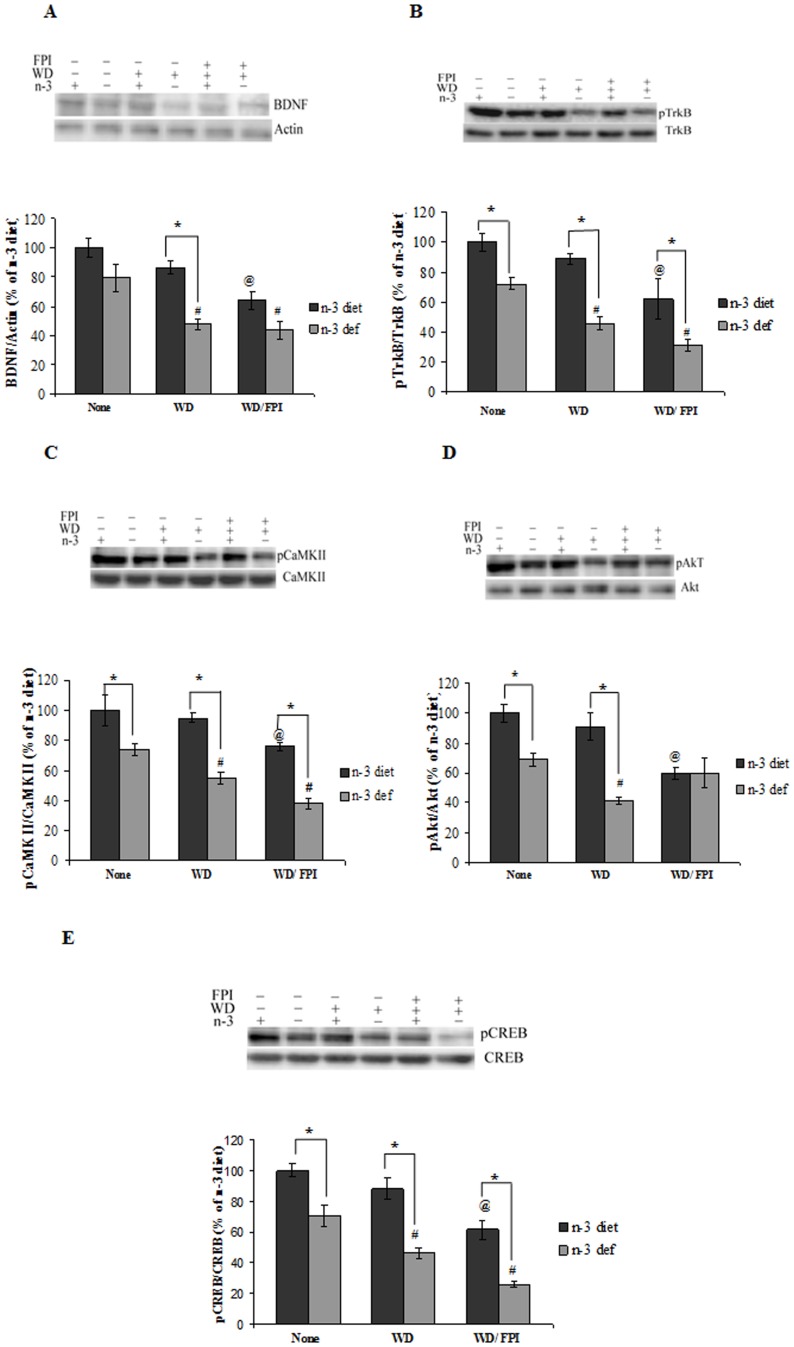
Effect of dietary transition and TBI on synaptic plasticity markers. Protein levels of (**A**) BDNF, (**B**) TrkB phosphorylation, (**C**) CaMKII phosphorylation, (**D**) Akt phosphorylation and (**E**) phosphorylation of CREB in groups fed with either omega-3 (n-3 diet) or omega-3 deficient (n-3 def) diet, switched to western diet (n-3 diet/WD and n-3 def/WD) and subjected to fluid percussion injury after diet transition (n-3 diet/WD/FPI and n-3 def/WD/FPI). Values are expressed as mean ± SEM. ^#^p<0.05 Vs n-3 def, ^@^p<0.05 Vs n-3 diet, ^*^p<0.05 Vs their respective n-3 diet group by ANOVA (two-way) and Newman–Keuls post-hoc test.

Brain-derived neurotrophic factor (BDNF) and its tropomyosin-related kinase receptor type B (TrkB) have a demonstrated role on neuronal plasticity and function [23]. There was a significant effect of diet (F_1,24_ = 27.95, p<0.01) and treatment (F_2,24_ = 17.01, p<0.01) for BDNF. Similarly, TrkB phosphorylation was significantly affected by diet (F_1,24_ = 37.96, p<0.01) and treatment (F_2,24_ = 17.09, p<0.01). The switching of n-3 deficient to WD decreased levels of BDNF and TrkB phosphorylation while the earlier exposure to n-3 diet preserved these levels. Exposure to TBI reduced levels of BDNF and its receptor beyond to that observed after switching to WD for the n-3 diet groups (Figure 4A); however, the effects on TrkB were more pronounced in WD rats previously exposed to the n-3 deficient diet (Figure 4B).

Through its TrkB receptor, BDNF activates Ca^2+^-calmodulin dependent kinase, CaMKII and Akt pathways with subsequent activation of cyclic AMP response element binding protein (CREB). A two-way ANOVA analysis (diet vs. treatment) showed a significant effect of diet (F_1,24_ = 68.01, p<0.01) and treatment (F_2,24_ = 17.32, p<0.01) on CaMKII. With regards to Akt phosphorylation, there were significant effects of diet (F_1,24_ = 23.40, p<0.01) and treatment (F_2,24_ = 7.158, p<0.01), as well as a significant interaction between diet and treatment effects (F_2,24_ = 6.898, p<0.01). We found that the dietary deficiency of n-3 resulted in significant decreases in CaMKII phosphorylation (Figure 4C) and Akt (Figure 4D), which were further aggravated by exposure to WD. In turn, prior exposure of n-3 diet attenuated the effects of WD on both CaMKII and Akt phosphorylation. Further, mTBI resulted in a significant decrease in CaMKII beyond to that observed after switching to WD, and these effects were more pronounced in the group originally exposed to the n-3 deficient diet. The phosphorylation of CREB was affected by diet (F_1,24_ = 69.74, p<0.01) and treatment (F_2,24_ = 31.61, p<0.01). Exposure to the n-3 deficient diet reduced CREB phosphorylation, and switching to WD aggravated this effect while subsequent TBI aggravated the effects even more (Figure 4E).

### Association between Synaptic Plasticity and Anxiety-like behavior

**Figure 5:**
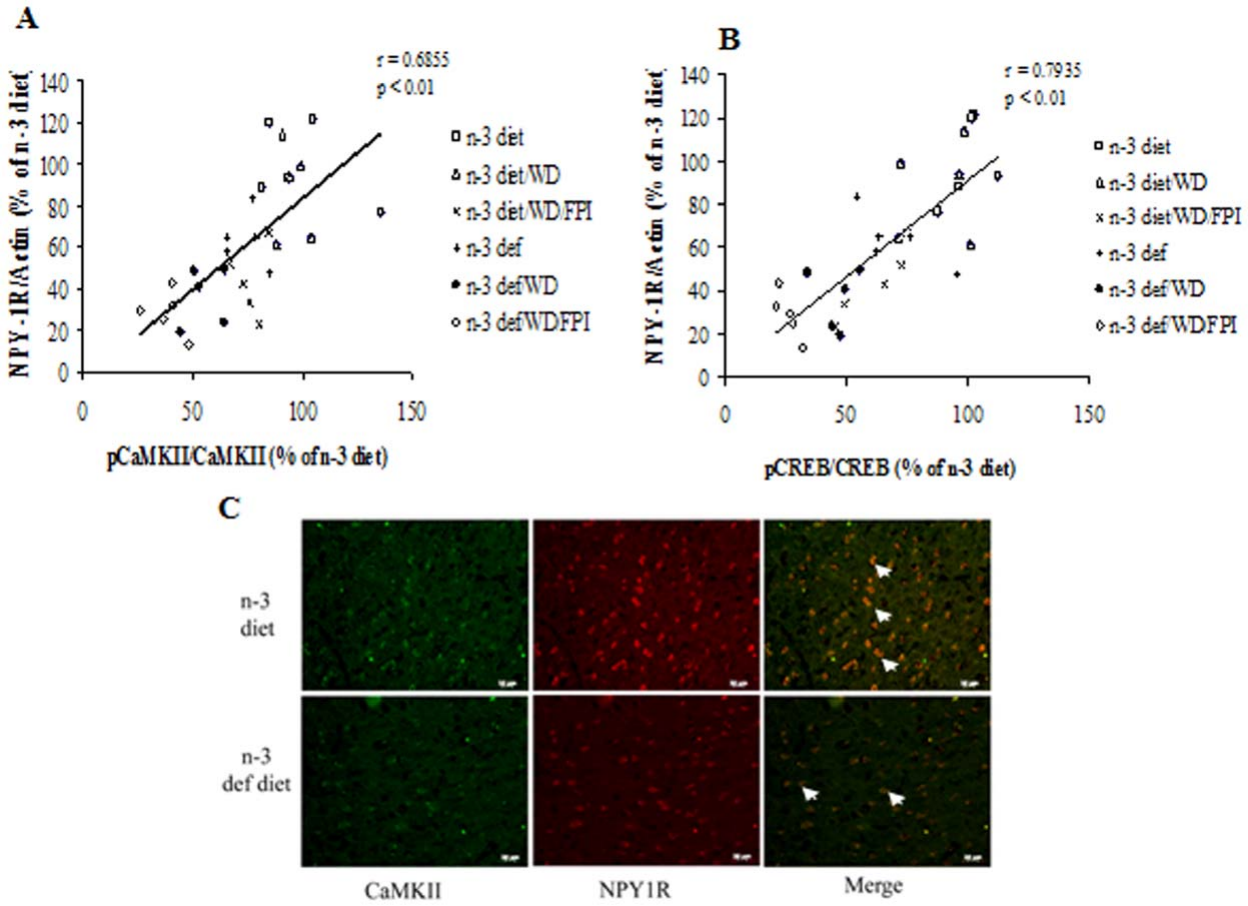
Association between molecules related with synaptic plasticity and anxiety-like behavior. Correlation analysis revealed a positive correlation between phosphorylated CaMKII and level of NPY1R (**A**) and phosphorylated CREB and level of NPY1R (**B**). (**C**) Immunoflouroscent staining for CaMKII and NPY1R proteins in coronal sections of frontal cortex region from omega-3 (n-3 diet) and omega-3 deficient (n-3 def) diet animals. Positive staining (green for CaMKII, red for NPY1R and yellow for co-stained cells, scale bar: 20 µm).

There was a positive correlation between phosphorylated CaMKII and NPY1R (r = 0.6855, p<0.01) suggesting that these parameters change in a coordinated fashion (Figure 5A). Furthermore, we found that the phosphorylated CREB varied in proportion to the NPY1R (r = 0.7935, p<0.01), which suggest that CREB activation is also coordinated with changes in NPY1R (Figure 5B).

Additionally, we analyzed the effects of n-3 diet on the phenotypic expression of CaMKII and NPY1R using immunofluorescence (Figure 5C). CaMKII and NPY1R labeling was apparent in neuronal cells that were distributed sparsely in the frontal cortex of n-3 deficient rats. The n-3 diet promoted a qualitative increase in the intensity in CaMKII and NPY1R co-stained cells in the frontal cortex (white arrows; Figure 5C).

### Immune Markers and Brain Challenges

**Figure 6:**
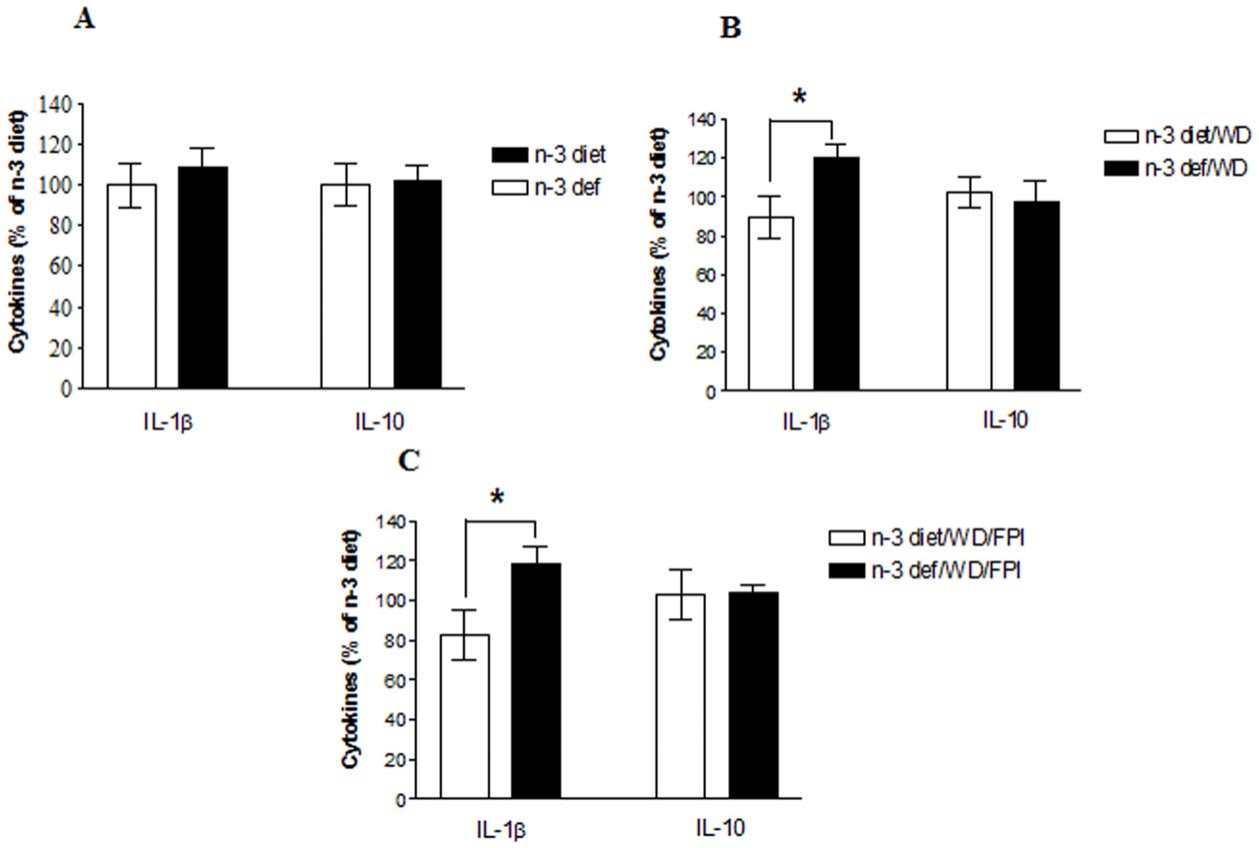
Effect of diet alteration and TBI on brain cytokines. Levels of IL-1β and IL-10 in groups fed with omega-3 (n-3 diet) or omega-3 deficient (n-3 def) diet (**A**), transitioned to western diet (n-3 diet/WD and n-3 def/WD) (**B**) and subjected to fluid percussion injury after diet transition (n-3 diet/WD/FPI and n-3 def/WD/FPI) (**C**). Values are expressed as mean ± SEM. *p<0.05 Vs their respective n-3 diet group by student’s (unpaired) t test.

**Figure 7:**
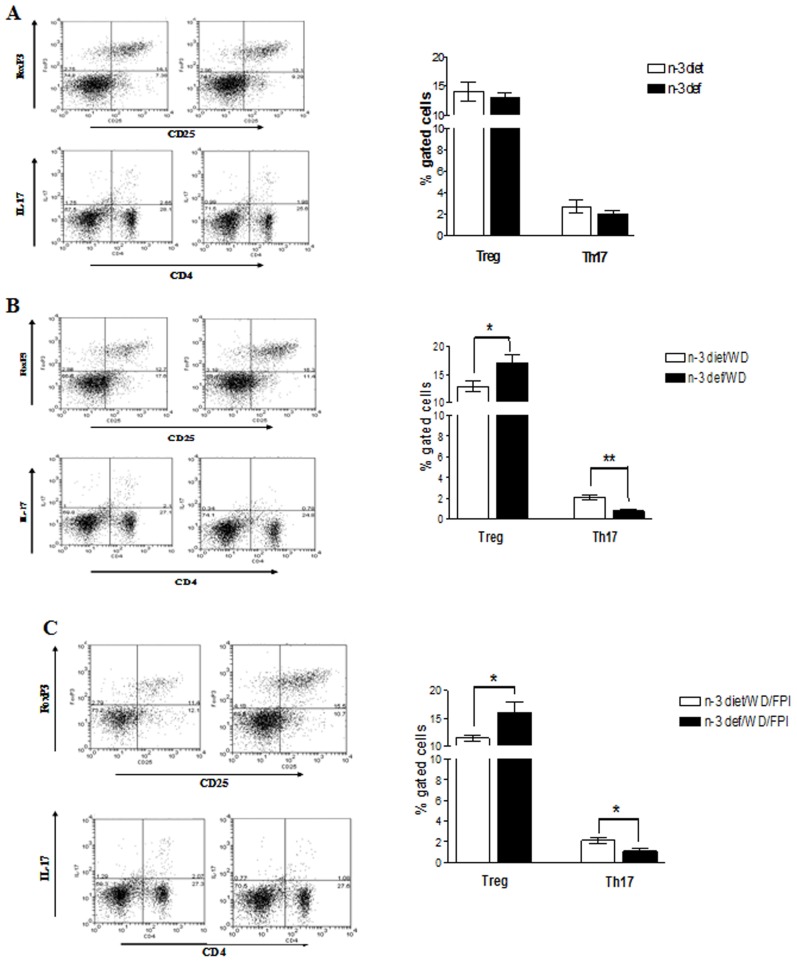
Immune markers affected by diet transition and brain trauma. Profiles of Treg cells and Th17 cells in groups fed with omega-3 (n-3 diet) or omega-3 deficient (n-3 def) diet (**A**), transitioned to western diet (n-3 diet/WD and n-3 def/WD) (**B**) and subjected to fluid percussion injury after diet transition (n-3 diet/WD/FPI and n-3 def/WD/FPI) (**C**). Plots for Treg cells represent percentage of CD4+ cells that are FoxP3+ and CD25+. Values are expressed as mean ± SEM. *p<0.05, **p<0.01 Vs their respective n-3 diet group by student’s (unpaired) t test.

We analyzed levels of IL-1β, a proinflammatory cytokine and IL-10, an anti inflammatory cytokine in the frontal cortex region of brain to assess the effects of diet on the inflammatory response in the CNS following TBI. Although n-3 deficiency alone had no effect on either cytokine (t_8_ = 0.6466, p>0.05; t_8_ = 0.1296, p>0.05) (Figure 6A), the switching of n-3 deficient diet to WD (t_8_ = 2.321, p<0.05) (Figure 6B) and/or TBI (t_8_ = 2.361, p<0.05) (Figure 6C) initiated an increase only in IL-1β. There were no changes in IL-10 levels after any of the treatment (t_8_ = 0.3762, p>0.05; t_8_ = 0.056, p>0.05). To assess the influence of the dietary interventions and TBI on the peripheral immune system we analyzed the abundance of Th subsets and regulatory T cells (Tregs) obtained from spleen by flow cytometry. Based on the pro or anti inflammatory activities, T cells have been classified as Th1, Th17 or Treg cells. Whereas Th1 and Th17 subsets release proinflammatory cytokines, regulatory T cells (Tregs) are known to suppress T cell proliferation and/or activity. In order to characterize an ongoing immune response, abundance of Th1, Th17 and Tregs (CD4+ CD25+ FOXP3+) were measured. Neither the n-3 adequate nor n-3 deficient diets by themselves had the ability to influence any of the T cell types (t_8_ = 0.5547, p>0.05; t_8_ = 1.066, p>0.05) (Figure 7A). However, the switching to the WD elicited a significant increase in Treg cells (t_8_ = 2.484, p<0.05) and a decrease in Th17 cells (t_8_ = 4.512, p<0.01) (Figure 7B) in the group of animal previously exposed to the n-3 deficient diet. TBI elicited a similar pattern of increase in Treg cells (t_8_ = 2.310, p<0.05) and decrease in Th17 cells (t_8_ = 2.563, p<0.05) in the n-3 deficient group transitioned to the WD (Figure 7C). On the other hand, the abundance of Th1 subsets was not significantly altered by any of the experimental manipulation (data not shown).

Nonetheless, the enhancement of Th17 cells and reduction of Tregs suggest the interesting possibility that the n-3 deficiency acts to predispose the immune system to a high state of activation in response to challenges imposed by the WD or TBI.

### Fatty Acid Levels in Brain

**Figure 8:**
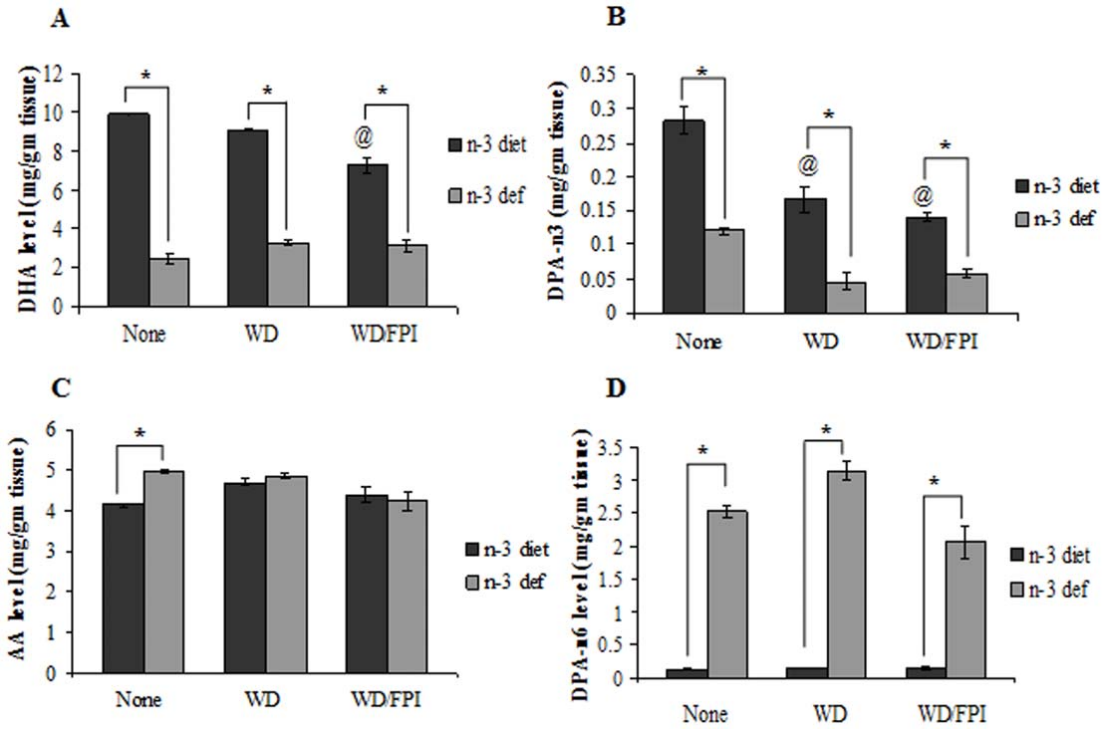
Fatty acid contents in cortical tissues. (A) Docosahexaenoic acid (DHA; C22:6*n*-3) (B) arachidonic acid (AA; 20:4*n*-6), (C) docosapentanoic acid-n3 (DPAn-3; 22:5*n*-3) and (D) docosapentanoic acid-n6 (DPAn-6; 22:5*n*-6) levels in groups fed with either omega-3 (n-3 diet) or omega-3 deficient (n-3 def) diet, switched to western diet (n-3 diet/WD and n-3 def/WD) and subjected to fluid percussion injury after switching of diet (n-3 diet/WD/FPI and n-3 def/WD/FPI). Values are expressed as mean ± SEM.^ #^p<0.05 Vs n-3 def, ^@^p<0.05 Vs n-3 diet, ^*^p<0.05 Vs their respective n-3 diet group by ANOVA (two-way) and Newman–Keuls post-hoc test.

We used gas chromatography to assess the effects of the dietary interventions on contents of polyunsaturated fatty acids (PUFAs) docosahexaenoic acid (DHA; 22:6*n*-3), arachidonic acid (AA; 20:4*n*-6), docosapentanoic acid (DPAn-3; 22:5*n*-3 and DPAn-6; 22:5*n*-6) in cortex region of the brain, as shown in Figure 8. A two-way ANOVA analysis (diet vs. treatment) indicated the effects of diet or treatment on DHA level (diet: F_1,24_ = 806.6, p<0.01; treatment: F_2,24_ = 9.903, p<0.01, and an interaction of diet and treatment: F_2,24_ = 21.13, p<0.01). With regards to DPAn-3 level, there were significant effects of diet (F_1,24_ = 138.2, p<0.01) and treatment (F_2,24_ = 40.61, p<0.01), as well as a significant interaction between diet and treatment effects (F_2,24_ = 4.74, p<0.05). The n-3 fatty acids DHA and DPAn-3 concentration was significantly lower in all the n-3 deficient diet groups. Alone WD transition significantly lowered the levels of DPAn-3 in n-3 adequate groups whereas the combination of transitioned WD and TBI significantly reduced both DPAn-3 and DHA levels in the n-3 fed animals. It appears that WD transition alone or followed by brain trauma were not effective to further reduce the already lowered levels of DHA and DPAn-3 in n-3 deficient groups (Figure 8A and B). A two-way ANOVA analysis (diet vs. treatment) showed a significant effect of diet (AA, F_1,24_ = 6.161, p<0.05; DPAn-6, F_1,24_ = 574.8, p<0.01) and treatment (AA, F_2,24_ = 5.963, p<0.01; DPAn-6, F_2,24_ = 9.331, p<0.01), as well as a significant interaction between diet and treatment effects (AA, F_2,24_ = 6.128, p<0.01; DPAn-6, F_2,24_ = 9.25, p<0.01) on n-6 fatty acid levels. When analyzed the major n-6 components in the brain of animals, the deficiency of n-3 in diet significantly induced the DPAn-6 in all the deficient groups whereas the significant induction in AA was observed only in alone n-3 deficient groups (Figure 8C and D).

## Discussion

We have assessed the role of diet as a vulnerability factor for the etiology of neurological and psychiatric disorders. We found that exposure to an n-3 diet during gestation and throughout maturation of the brain are crucial for building neural resilience during adulthood. The lack of dietary n-3 during brain maturation worsened the effects of transition to a WD and subsequent TBI on anxiety-like behavior and molecular counterpart. It also appears that the transition to WD and TBI in rats enhanced the state of inflammation in animals deprived of n-3 during development. According to our results, diet may have the capacity to influence substrates of neurological disorders by acting on key elements that underlie neuronal plasticity and cognitive processing.

### Anxiety-like behavior: Prior Exposure to Dietary DHA Influences the Consequences of WD on the Outcome of TBI

Growing evidence indicates that mild TBI can trigger neuropsychological imbalances characterized by anxiety and cognitive inability, and is considered a risk factor for PTSD [24]. The concussion paradigm used in our study, involving minimal neuronal death in spite of cognitive and emotional detriment [25], has been thought to be a suitable rat model for mechanistically studying PTSD [13]. The causes of PTSD are undetermined while assumingly multifactorial, in which the individual’s lifestyle likely plays a major role. Our results showed that effects of mTBI were more pronounced in the n-3 deficient animals switched to WD, arguing about the critical role of dietary n-3 to protect against the development of anxiety like disorders after TBI. In our previous studies, we have reported the protective influence of dietary DHA to improve cognitive impairment following brain trauma in rodents [8], [9].

The neuropeptide Y1 receptor (NPY1R) is a G-protein coupled receptor responsible for signaling the anxiolytic effects of NPY [22] and is a putative marker of PTSD [26]. Our results showed that either the lack of n-3, WD transition, and/or mTBI strongly attenuated NPY1R levels, or there was a strong association between lowered NPY1R and anxiety-like behavior. In turn, adequate levels of n-3 fatty acids appeared to protect against the effects of WD and mTBI, and these effects were reflected in higher levels of NPY1R. These results support the possibility that reductions in NPY1R levels may contribute to observed changes in anxiety-like behavior. This interpretation is in general agreement with studies showing stress resilience in rats over expressing NPY [27], or increased susceptibility to stress and anxiety in NPY knockout mice [28]. Based on findings that NPY is localized to brain regions relevant to PTSD and that NPY levels are low in the cerebrospinal fluid of combat veterans diagnosed with PTSD [26], ours results provide insight to better understand how dietary habits can influence the pathobiology of PTSD.

### Molecules Associated with Synaptic Plasticity

Disruption in BDNF function has been implicated in the pathophysiology of several neuropsychological disorders such as anxiety [29], depression [30] and schizophrenia [31]. Most treatments against anxiety and depression are associated with the action of BDNF [32]. Based on this information and on evidence that patients suffering PTSD have lower level of plasma BDNF [18], it is reasonable to assume that dysfunction in the BDNF system is a risk factor for the pathobiology of PTSD. According to our results, conditions of n-3 deficiency, WD and mTBI reduced BDNF levels or BDNF-TrkB signaling, while dietary n-3 showed to offset these effects.

According to our results, the effects of diet and subsequent TBI appeared to concert the actions of key elements in the BDNF signaling such as TrkB, CREB, CaMKII, and Akt, in association with NPY1R. In particular, we found a positive correlation between levels of NPY1R Vs CREB and Vs CaMKII, and the cellular expression of NPY1R was co-localized with that of CaMKII. CREB is an important step in BDNF signaling and a point of convergence of signaling pathways regulating synaptic activity and anxiety [33] such that the effects of n-3 increasing CREB activation may be significant for modulation of synaptic plasticity. NPY can also act through CAMKII to regulate CREB phosphorylation [34], and this interaction may be reflected by our results showing that levels of NPY1R change in proportion to levels of CAMKII and CREB.

### “Metabolic Programming” as a Source of Vulnerability for Neuropsychological Disorders

The concept of “metabolic programming” was coined to describe the action of a nutritional stress/stimulus during critical periods of early development on altering an organism’s physiology and metabolism much later in life [35]. Although the consequences of “metabolic programming” are suspected to be critical for brain function, its molecular mechanisms are poorly understood. Our results showing that the n-3 diet deficiency during brain formation and subsequent transition to WD altered brain capacity to sustain insults during adulthood, add clues to understand the molecular basis of metabolic programming. DHA is a component of plasma membranes and crucial for proper signaling of embedded transmembrane receptors, and highly susceptible to metabolic regulation [36]. Reduced brain levels of n-3 (DHA and DPAn-3) and increased levels of n-6 (AA and DPAn-6) found in the animal group exposed to deficient n-3 may have impaired neuronal signaling and contributed to the protracted molecular and behavioral plasticity in adulthood. The reduction observed in n-3 fatty acids after TBI is might be due to the combined influence of WD transition and TBI. The WD used has high contents of sugar and total fats that may have contributed to reduce brain plasticity as indicated by recent studies [36] showing that chronic sugar consumption can lead to protracted plasticity, particular under deficiency of n-3 fatty acids.

The fact that the actions of the BDNF system on synaptic plasticity and behavior are closely associated with energy regulation [4], portray BDNF as a key player in the mechanisms of “metabolic programming”. Further studies are necessary to define how the interaction between BDNF, metabolism and the epigenome are central for the effects of environmental factors on the etiology of many neurological and psychiatric disorders.

### Brain Challenges Stimulate the Immune System

It is becoming recognized that the immune system is an important mediator for the effects of environmental stimuli on the brain, particularly in the pathobiology of PTSD [37]. According to our results, n-3 feeding did not affect cytokine levels in frontal cortex or T-cells in the spleen of rats that did not receive any challenge. It appears that the absence of n-3 in the diet may predispose the reaction of the immune system to other insults such as change in the diet or TBI. This is evidenced by the enhanced levels of IL-1β in frontal cortex, reductions of Th 17 cells in spleen, and increase of the immune suppressive cell type (Treg) in the spleen after switching to WD and/or mTBI. Although a variety of effects of n-3 fatty acids and WD on Tregs in vitro and in vivo have been described [38], the effects of dietary changes and/or injury have not been investigated. The effects of reduced n-3 dietary intake, on what appeared to be a state of higher immune activity following dietary or injury challenge is a novel finding. One possible explanation for our findings is that n-3 deficiency during development may induce a latent state of immune alertness that is expressed later on in life by the challenges imposed by dietary switching or injury. In any case, the studies demonstrate that dietary factors during the developmental period are an environmental variable that can have long-term consequences on behavioral and immune responses, particularly under challenging conditions. Our results are in general agreement with the studies displaying an increment of CD4^+^ CD25^+^ T regulatory cell activity in trauma patients while depressing the Th1 immunity [39]. This implies that the increase in Tregs found in rats with n-3 deficiency has the potential to suppress any-injury induced T cell activation. These results have important implications for the understanding how environmental challenges perturb innate immunity, and predispose the brain to the effects of neurological challenges in adult life.

### Conclusions

**Figure 9:**
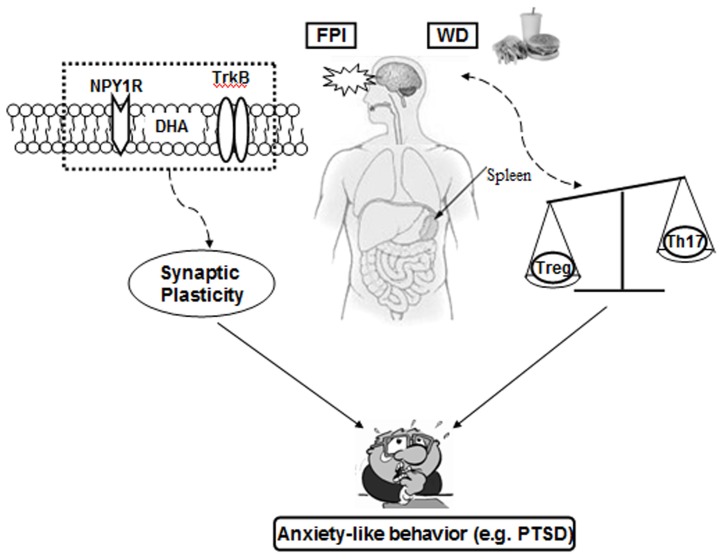
A proposed model for how diet influences anxiety-like behavior after traumatic brain injury (TBI), and its implications for post-traumatic stress disorder (PTSD). The contents of n-3 during gestation and early life influence the vulnerability of the brain to future challenges (changes to western diet (WD) or TBI) during adult life. Dietary n-3 is crucial for the structure and function of the plasma membrane influencing neuronal signaling of embedded receptors such as the BDNF receptor TrkB or the NPY receptor. Transition from n-3 deficient to WD increases the vulnerability via suppressing NPY1R and TrkB signaling. These alterations can result in abnormal neuronal signaling and associated behaviors such as anxiety. Dietary transition with traumatic brain injury may also affect the immune system during n-3 deficiency that may interact with pathophysiologic domain relevant to mood regulation. Overall, the proposed mechanism is important to understand how dietary transition can regulate the vulnerability of the adult brain to develop PTSD following traumatic brain injury.

Our results emphasize the powerful action of diet during early life for determining later susceptibility to brain insults, involving elements associated with plasma membrane signaling, synaptic plasticity, and immune system. These results seem to provide molecular basis for the poorly understood action of metabolic programming on the regulation of neuropsychological disorders in adult life (Figure 9). Given the increasing consumption of unhealthy diets in environments with high prevalence of brain trauma [7], diet may be a factor for predisposing towards the development of disorders like PTSD. However, further studies are necessary to document how diet and TBI can interact to influence the complexity of PTSD-like disorders. These results can apply to better understand the course of the TBI pathobiology in individuals exposed to head trauma such as sport athletes, military personnel as well as victims of domestic violence. These results provide novel evidence that can be used for designing strategies to reduce vulnerability to neurological disorders based on the strong and non-invasive capacity of dietary factors to influence neuronal function and plasticity.
